# Choroid plexus immune cell response in murine hydrocephalus induced by intraventricular hemorrhage

**DOI:** 10.1186/s12987-024-00538-4

**Published:** 2024-04-23

**Authors:** Yingfeng Wan, Xiongjie Fu, Tianjie Zhang, Ya Hua, Richard F. Keep, Guohua Xi

**Affiliations:** 1 Department of Neurosurgery, University of Michigan, Ann Arbor, MI USA; 2 R5018 Biomedical Science Research Building, University of Michigan, 109 Zina Pitcher Place, 48109-2200 Ann Arbor, MI USA

**Keywords:** Hydrocephalus, Intraventricular hemorrhage, iron, Peroxiredoxin 2, Choroid plexus, Immune cells, Macrophages, Lymphocytes, Neutrophils

## Abstract

**Background:**

Intraventricular hemorrhage (IVH) and associated hydrocephalus are significant complications of intracerebral and subarachnoid hemorrhage. Despite proximity to IVH, the immune cell response at the choroid plexus (ChP) has been relatively understudied. This study employs CX_3_CR-1^GFP^ mice, which marks multiple immune cell populations, and immunohistochemistry to outline that response.

**Methods:**

This study had four parts all examining male adult CX_3_CR-1^GFP^ mice. Part 1 examined naïve mice. In part 2, mice received an injection 30 µl of autologous blood into right ventricle and were euthanized at 24 h. In part 3, mice underwent intraventricular injection of saline, iron or peroxiredoxin 2 (Prx-2) and were euthanized at 24 h. In part 4, mice received intraventricular iron injection and were treated with either control or clodronate liposomes and were euthanized at 24 h. All mice underwent magnetic resonance imaging to quantify ventricular volume. The ChP immune cell response was examined by combining analysis of GFP(+) immune cells and immunofluorescence staining.

**Results:**

IVH and intraventricular iron or Prx-2 injection in CX_3_CR-1^GFP^ mice all induced ventriculomegaly and activation of ChP immune cells. There were very marked increases in the numbers of ChP epiplexus macrophages, T lymphocytes and neutrophils. Co-injection of clodronate liposomes with iron reduced the ventriculomegaly which was associated with fewer epiplexus and stromal macrophages but not reduced T lymphocytes and neutrophils.

**Conclusion:**

There is a marked immune cell response at the ChP in IVH involving epiplexus cells, T lymphocytes and neutrophils. The blood components iron and Prx-2 may play a role in eliciting that response. Reduction of ChP macrophages with clodronate liposomes reduced iron-induced ventriculomegaly suggesting that ChP macrophages may be a promising therapeutic target for managing IVH-induced hydrocephalus.

**Supplementary Information:**

The online version contains supplementary material available at 10.1186/s12987-024-00538-4.

## Introduction

Intraventricular hemorrhage (IVH) frequently occurs after intraparenchymal or subarachnoid hemorrhage (SAH). Thus, 30–50% of intracerebral hemorrhage (ICH) patients exhibit intraventricular extension, and nearly half of these cases develop hydrocephalus [1]. Currently, there are no pharmacological interventions approved for treating post-hemorrhagic hydrocephalus (PHH) [2]. It is typically managed neurosurgically with cerebrospinal fluid (CSF) diversion (shunt placement or endoscopic third ventriculostomy).

Blood components contribute to secondary brain injury after cerebral hemorrhage including IVH. Iron, a product from the lysis of erythrocytes and the subsequent degradation of hemoglobin, contributes to hydrocephalus development in rats [3, 4]. Peroxiredoxin-2 (Prx-2) is the third most common protein in red blood cells. Extracellular Prx-2 acts as a proinflammatory damage-associated molecular pattern (DAMP), facilitating the release of proinflammatory factors by microglia/macrophage [5, 6]. Intraventricular injection of Prx-2 induces hydrocephalus and the effects of IVH can be attenuated by the administration of Conoidin A, a Prx-2 inhibitor [7, 8].

While the role of inflammation in PHH pathogenesis has been widely studied [2], there has been limited focus on the choroid plexus (ChP) immune cell response following IVH. This is despite the proximity of the ChP to the IVH and a growing understanding of the role of the ChP in neuroinflammatory conditions [9, 10] and a potential role of ChP inflammation in regulating CSF production [11, 12]. Consequently, there is a critical need to explore the pathophysiological mechanisms of inflammation at the ChP in both IVH and PHH.

The central nervous system (CNS) hosts a distinct population of immune cells known as border-associated macrophages (BAMs), which encompass meningeal macrophages, perivascular macrophages and ChP macrophages [13]. Recent research has shed light on the role of meningeal and perivascular macrophages in various neurological diseases, including SAH and ischemic stroke [14, 15]. Our prior publications have underscored the potential involvement of ChP macrophages in the pathogenesis of hydrocephalus. Depletion of ChP stromal macrophages reduced the hydrocephalus induced by iron [3] and Prx-2 [8]. Furthermore, intraventricular injection of Prx-2 [7] and thrombin [16] resulted in ChP neutrophil accumulation. However, our understanding of the PHH-induced changes in ChP immune cells (including epiplexus macrophages, stromal macrophages, dendritic cells, natural killer (NK) cells, lymphocytes and neutrophils), remains limited.

Liposomes, which are lipid vesicles synthetically created, serve as carriers for encapsulating hydrophilic drug molecules, including clodronate. Macrophages have the capacity to internalize clodronate liposomes, a process that ultimately leads to their own cell death [17]. Depleting macrophages via clodronate liposomes has been employed as a strategy in various animal models, including ICH [18], ischemic stroke [19], allergic encephalomyelitis [20], and spinal cord injury [21]. While some investigations have explored the potential mechanisms of clodronate liposomes in the context of blood component-induced hydrocephalus in rats [3, 8], there remains a limited understanding of the therapeutic effects and underlying mechanisms of clodronate in the PHH. Further research is required to fully elucidate these aspects.

CX_3_CR-1^GFP^ mice are characterized by a knock-in/knock-out design that leads to the expression of enhanced green fluorescent protein (EGFP) in monocytes, dendritic cells, NK cells and microglia under the control of the endogenous CX_3_CR1 locus. These mice have been used to examine leukocyte migration and trafficking [22] and the immune cell response to ischemic stroke, spinal cord injury, traumatic brain injury, laser burn and maternal immune activation [23–27]. However, to date, there has been no documented utilization of CX_3_CR-1^GFP^ mice as a resource for investigating the response of ChP immune cells following IVH and the subsequent PHH development.

The current study used CX_3_CR-1^GFP^ mice to examine activation and alterations in ChP immune cells after hydrocephalus induced by IVH or blood components (iron and Prx2). Additionally, it evaluated the impact of clodronate liposome treatment on hydrocephalus and its effects on ChP immune cells. Clodronate liposomes kill macrophages after internalization [17].

## Materials and methods

### Animal preparation and intraventricular injection

The University of Michigan Committee on the Use and Care of Animals approved the animal use protocols. CX_3_CR-1^GFP^ mice were purchased from Jackson Laboratory (Stock No: 005582, Bar harbor, ME, United States). A total of 45 male CX_3_CR-1^GFP^ mice aged of 2–3 months were used. Animals were anesthetized with ketamine (90 mg/Kg IP) and xylazine (5 mg/Kg IP) and body temperature maintained at 37.5 °C with a heating pad. The right femoral artery was catheterized to obtain blood for injection without anticoagulant. In a stereotactic frame (Kopf Instruments, Tujunga, CA), a cranial burr hole (1 mm) was drilled and a 26-gauge needle stereotactically inserted into the right lateral ventricle (coordinates: 0.5 mm posterior, 2.6 mm ventral, and 1.1 mm lateral to the bregma). A micro-infusion pump (World Precision Instruments Inc., Sarasota, FL) was used for intraventricular injections (3 µl/min). The needle was left in place for an additional ten minutes after the injection, the burr hole sealed with bone wax and the skin incision sutured closed.

### Experimental groups

This study was subdivided into four parts with animals randomized into each group. First, naive CX_3_CR-1^GFP^ mice were euthanized after magnetic resonance imaging (MRI) (*n* = 6). Second, CX_3_CR-1^GFP^ mice underwent intraventricular injection of autologous blood (30 µl, *n* = 7) into the right lateral ventricle at a rate of 3 µl/min. At 24 h after injection, mice underwent MRI and were euthanized. Third, CX_3_CR-1^GFP^ mice underwent intraventricular injection of saline (12.5 µl, *n* = 6) or FeCl_3_ (0.5mmol/L, 12.5 µl, *n* = 7) or Prx2 (1 mg/ml, 12.5 µl, *n* = 7) into the right lateral ventricle at a rate of 3 µl/min. We injected 12.5 µl of blood components (FeCl_3_, Prx-2) to mimic the approximate volume of erythrocytes in a 30 µl whole blood injection. 24 h after injection, mice underwent MRI and were euthanized. Fourth, CX_3_CR-1^GFP^ mice underwent intraventricular injection of FeCl_3_ + clodronate liposome (final concentration 0.5 mmol/L, 3.5 g/L respectively, 12.5 µl, *n* = 6) or FeCl_3_ + control liposome (final concentration 0.5 mmol/L, 3.5 g/L respectively, 12.5 µl, *n* = 6) into the right lateral ventricle. The liposome concentration used in this study is based on our previous studies and this concentration can reduced hydrocephalus in rat [3, 8]. 24 h after injection, mice underwent MRI and were euthanized. In all experiments, brains were used for immunohistochemistry. Our decision to use only male mice in this study was driven by the need to eliminate hormonal variations that could affect the hydrocephalus and the subsequent immune cells response in the choroid plexus. This choice was informed by our previous study, which found that female rats have more severe ventricular dilation compared to male rats [16]. We chose the 24-hour timepoint because 24 h after IVH is critical for observing the peak effects of hydrocephalus being studied. Our prior research indicated that hydrocephalus peaks at 24 h after IVH in rats [28].

### MRI scanning and ventricle volume measurement

For MRI scanning, mice were anesthetized with 2% isoflurane. MRI was obtained in a 7.0-T Varian MR scanner (Bruker Inc.) with a T2 fast spin-echo sequence (TR/TE = 4000/60 msec). The field of view was 35 mm × 35 mm, and the matrix was 256 mm×128 mm. Twenty-five coronal slices (0.5 mm thick) were acquired to cover the lateral ventricle system. Ventricular volumes were calculated as previously described [29]. Briefly, bilateral lateral ventricles were outlined and the area on each slice was measured. Ventricular volume was calculated by multiplying the ventricular area by section thickness. A blinded observer performed the image analyses using Image J.

### Immunofluorescence staining

Mice were euthanized with pentobarbital (100 mg/kg intraperitoneal) and underwent transcardiac perfusion with 4% paraformaldehyde in 0.1 mol/L phosphate-buffered saline (pH 7.4). Brains were stored in 4% paraformaldehyde for one day followed by immersion in 30% sucrose for 3 days at 4 °C. They were then embedded in optimal cutting temperature compound (Sakura Finetek USA) prior to sectioning (18-µm) on a cryostat. Immunofluorescence studies were performed as previously described [3]. Briefly, primary antibodies were added and incubated overnight at 4 °C followed by a phosphate buffered saline wash and incubation at room temperature for 2 h with secondary antibodies. Primary antibodies were polyclonal rabbit anti-Iba-1 (019-19741, 1:200 dilution; Wako), monoclonal rat anti-CD4 (100,506, 1:200 dilution; BioLegend), monoclonal rabbit anti-beta catenin (ab16051, 1:200 dilution; Abcam), polyclonal rabbit anti-NKCC1 (ab59791, 1:200 dilution; Abcam), monoclonal rat anti-MHC class II (I-A/I-E) (17-5321-82, 1:200 dilution; Invitrogen), monoclonal rat anti-mouse CD31 (557,355, 1:200 dilution; BD Biosciences), and polyclonal goat anti-myeloperoxidase (MPO; AF3667, 1:200 dilution; R&D systems). Negative controls were executed by eliminated primary antibody. Secondary antibodies were Alexa Fluor 594 donkey anti-rat IgG (1:500, Invitrogen), Alexa Fluor 594 donkey anti-goat IgG (1:500, Invitrogen), Alexa Fluor 594 donkey anti-mouse IgG (1:500, Invitrogen), and Alexa Fluor 594 donkey anti-rabbit IgG (1:500, Invitrogen). Fluoroshield™ with DAPI (F6057) was used for nuclear labeling. Details on antibody specificity are given in Supplemental Fig. 1.

### Cell counting and soma size measurement

Immune-associated cells were assessed on high-power images (x40 magnification) taken by a digital camera with attached to a microscope (Olympus Life Science) with UPLFLN semi-apochromat objectives. The brain section (∼ 0.4 mm posterior to the bregma) with two ChP in bilateral lateral ventricles was chosen to count cells. GFP positive cells, T lymphocytes and neutrophils as a percentage of total ChP cell number were calculated. We used NKCC1, a marker of the apical surface of choroid plexus epithelial cells, to distinguish between epiplexus and stromal macrophages. The percentage of macrophages, epiplexus macrophages and stromal macrophages were calculated as a percentage of total GFP positive cell number. Macrophages are GFP positive, allowing us to quantify macrophages by the total GFP (+) cells. However, the majority of T cells and neutrophils are GFP negative, so we did not quantify their number relative to total GFP (+) cells. Instead, we chose to quantify them relative to the total number of ChP cells. Ten GFP-positive cells from one sample were randomly chosen to have their soma size measured. We used Image J to outline the margins of the chosen cells and measured their soma size. The average number soma size of the ten selected cells were used in the statistical analysis. Cell counts and soma size measurements were performed using Image J. All measurements were repeated three times by a blinded observer and the mean value was used.

### Statistical analysis

Statistical analysis was performed using GraphPad Prism software (version 8.0, San Diego, California). Presence of a normal distribution was determined using the Kolmogorov–Smirnov test. Unpaired student t-test and one-way ANOVA with Tukey’s multiple comparisons test were used for data with a normal distribution and values are displayed as means ± standard deviation (SD). Mann-Whitney U-tests and nonparametric Kruskal-Wallis test with Dunn’s multiple comparisons test were used for data with a non-normal distribution and values are displayed as median (25th percentile, 75th percentile). Significant differences were considered as *p* < 0.05.

## Results

### Choroid plexus GFP(+) cells consist of a heterogeneous population comprising macrophages, dendritic cells and NK cells

**Fig. 1:**
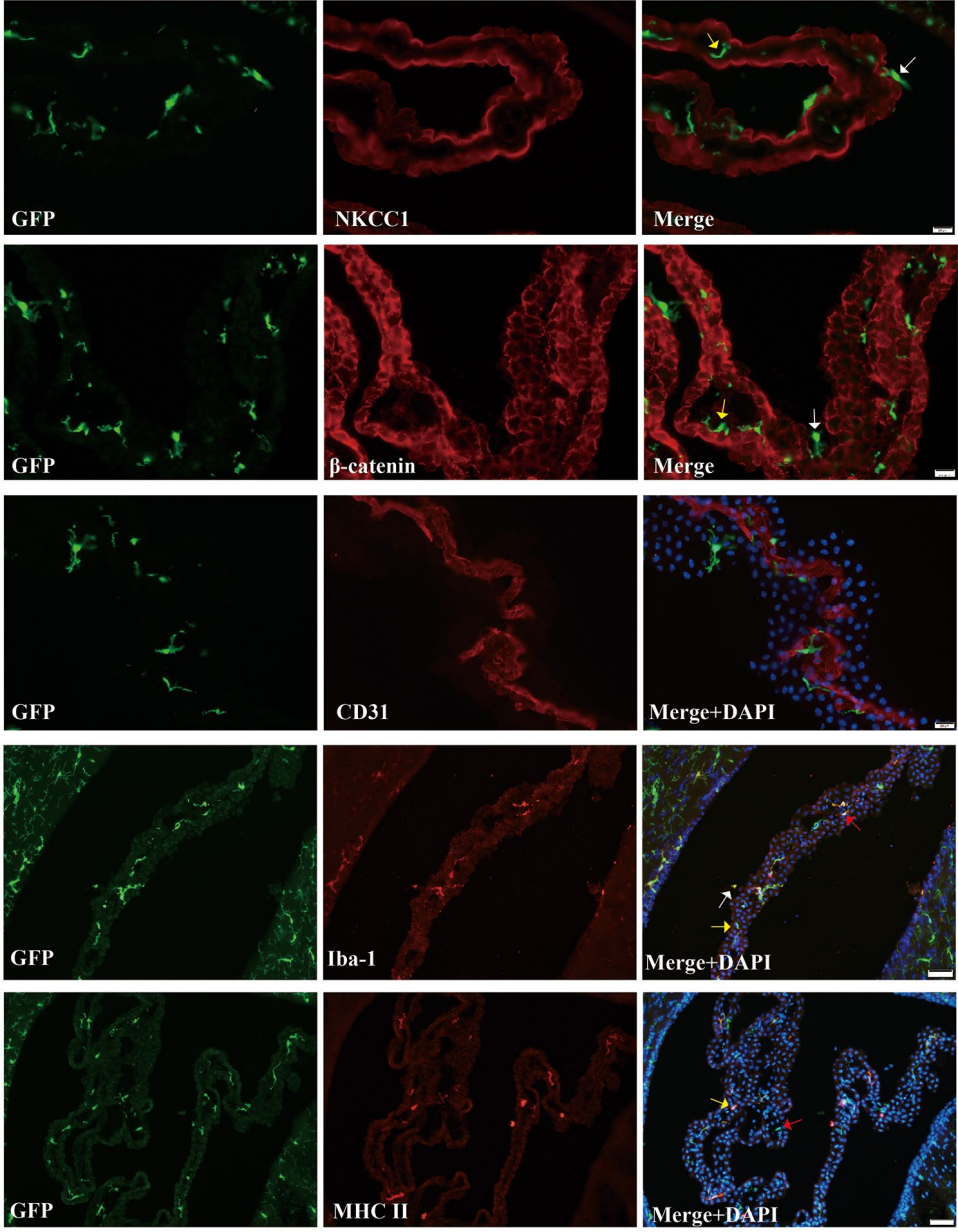
Baseline characteristics of choroid plexus (ChP) immune cells in naïve CX_3_CR-1^GFP^mice. First row: Immunofluorescence staining for NKCC1 (apical epithelial marker) in ChP of naïve CX_3_CR-1^GFP^ mice. Numerous green fluorescent protein (GFP)(+) cells were evident within the ChP stroma (located beneath the epithelium, indicated by the yellow arrow), while fewer GFP(+) cells were observed on the ChP epithelium apical surface (epiplexus cells; indicated by the white arrow, scale bar = 20 μm). Second row: Immunofluorescence staining for β-catenin (epithelial marker) in ChP of naïve CX_3_CR-1^GFP^ mice. Numerous green fluorescent protein (GFP)(+) cells were evident within the ChP stroma (located beneath the epithelium, indicated by the yellow arrow), while fewer GFP(+) cells were observed on the ChP epithelium apical surface (epiplexus cells; indicated by the white arrow, scale bar = 20 μm). Third row: Immunofluorescence staining for CD31 (blood vessel marker) in ChP of naïve CX_3_CR-1^GFP^ mice. No GFP(+) cells were detected in the ChP blood vessel. Scale bar = 20 μm. Fourth row: Immunofluorescence staining for Iba-1 in ChP of naïve CX_3_CR-1^GFP^ mice. The white arrow highlights epiplexus GFP(+)/Iba-1(+) cells, the red arrow indicates stromal GFP(+)/Iba-1(+) cells, and the yellow arrow signifies GFP(+)/Iba-1(-) cells (scale bar = 50 μm). Fifth row: Immunofluorescence staining for MHC II in ChP of naïve CX_3_CR-1^GFP^ mice. The yellow arrow indicates stromal GFP(+)/MHC II(+) cells, and the red arrow signifies GFP(+)/MHC II(-) cells (scale bar = 50 μm)

In naïve CX_3_CR-1^GFP^ mice, the ChP harbored several types of GFP positive (+) immune cells, exhibiting diverse cellular morphologies, including stellate and round shapes, as depicted in Fig. 1. The majority of these GFP(+) cells resided within the ChP stroma (indicated by the yellow arrow), with only sporadic occurrences of GFP(+) cells was observed on the apical surface of ChP epithelial cells (indicated by the white arrow). No GFP(+) cells were detected in ChP blood vessels.

At the ChP, a dense population of Iba-1(+) cells was distributed both within the stromal and apical regions of the epithelium (Fig. 1). For the purpose of this study, in line with the established definitions from previous research on ChP macrophages [3, 8], we categorize GFP(+)/Iba-1(+) cells as macrophages, while GFP(+)/Iba-1(-) cells are identified as dendritic cells and NK cells, as denoted by the yellow arrow. Additionally, we further classified ChP macrophages into epiplexus macrophages (indicated by the white arrow) and stromal macrophages (indicated by the red arrow), based on their position relative to the ChP epithelial cells.

A notable number of major histocompatibility complex II (MHC II) (+) cells was evident within the ChP stroma (Fig. 1). In our classification schema, we define ChP GFP (+)/MHC II (+) cells as encompassing both macrophages and dendritic cells (indicated by the yellow arrow). ChP GFP (+)/MHC II (-) cells are identified as NK cells (indicated by the red arrow).

### IVH-induced hydrocephalus, resulted in increased in numbers of ChP GFP(+) immune cells and enlarged cellular soma size in CX _3_CR-1^GFP^mice

**Fig. 2:**
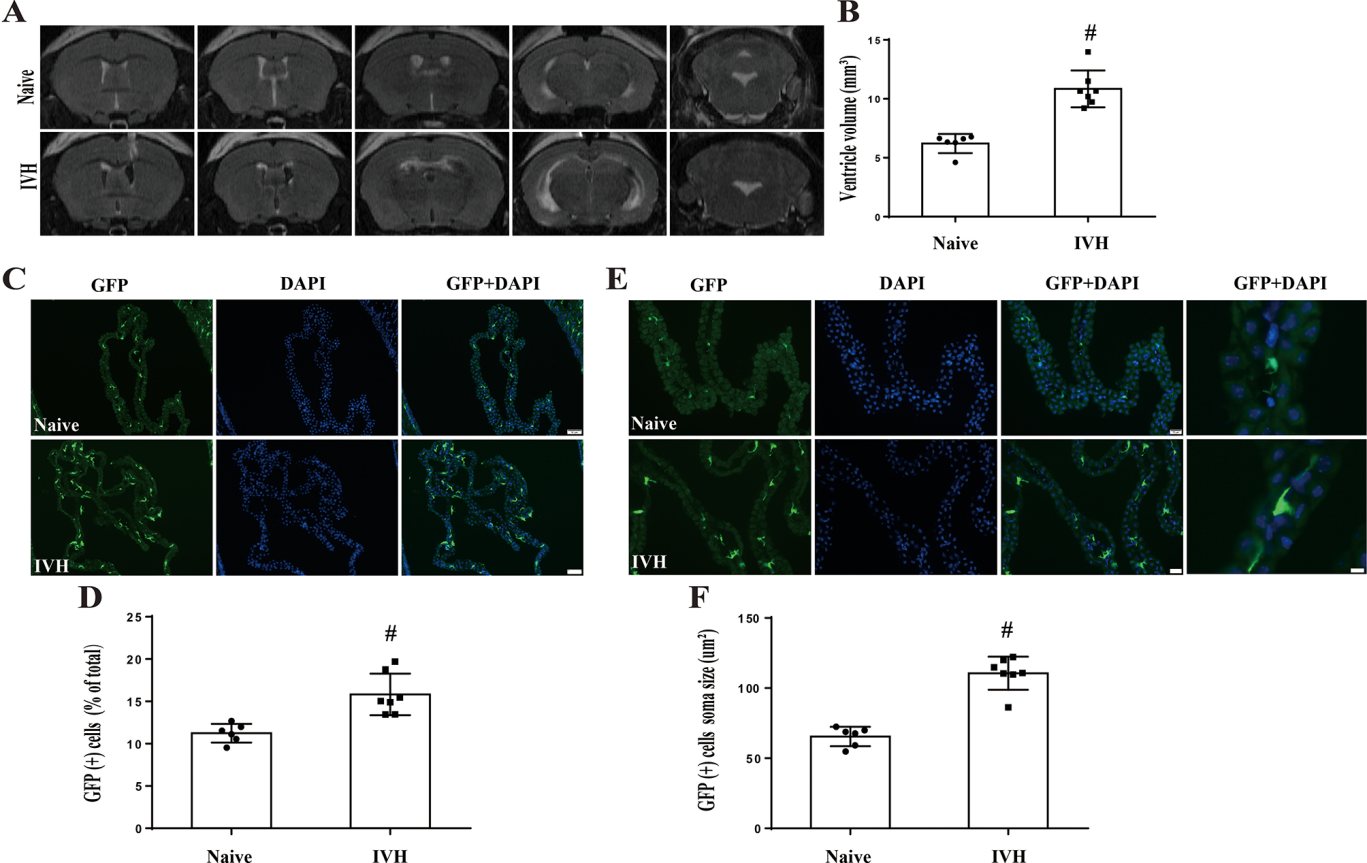
Intraventricular hemorrhage increases ventricular volume as well as the number of ChP GFP(+) cells and soma size of those cells in CX_3_CR-1^GFP^mice. (**A**) Examples of T2-weighted MRI scans comparing naïve CX_3_CR-1^GFP^ mice with CX_3_CR-1^GFP^ mice at 24 h post-IVH. Note the ventricular dilation and presence of blood within the ventricle in the IVH model. (**B**) Quantification of ventricular volume. Values are means ± SD, *n* = 6 in naive group, *n* = 7 in IVH group, #*p* < 0.01 by student *t*-test. (**C**) Fluorescence images of GFP(+) cells in the ChP of naïve and 24 h post-IVH mice. Scale bar = 50 μm. (**D**) The number of GFP(+) cells was calculated as a percentage of total ChP cells. Values are mean ± SD, *n* = 6 in naive and *n* = 7 in IVH groups. #*p* < 0.01 by student *t*-test. (**E**) Fluorescence images of GFP(+) cells in the ChP of naïve mice and 24 h post-IVH. The first columns scale bar = 20 μm and fourth column scale bar = 5 μm. (**F**) Soma sizes of ChP GFP(+) cells were quantified. Values are mean ± SD, *n* = 6 in naive and *n* = 7 in IVH groups. #*p* < 0.01 by student *t*-test

Figure 2A displays representative MRIs of both naïve mice and those with hydrocephalus caused by IVH. IVH increased ventricle volume (10.8 ± 0.5 mm^3^; *n* = 7) compared to naïve mice (ventricle volume 6.2 ± 0.3 mm^3^; *n* = 6, *P* < 0.01, Fig. 2B). IVH increased the number of GFP(+) cells at 24-hour timepoint (15.8 ± 0.9% of all ChP cells; *n* = 7) compared to naïve mice (11.2 ± 0.4%; *n* = 6, *P* < 0.01, Fig. 2C and D). Concurrently, IVH increase the soma size of ChP GFP(+) cells (111 ± 4 um^2^; *n* = 7) compared to naïve mice (65 ± 3 um^2^; *n* = 6, *P* < 0.01, Fig. 2E and F).

### Number of ChP macrophages (total and epiplexus), T lymphocytes and neutrophils increased following IVH

**Fig. 3:**
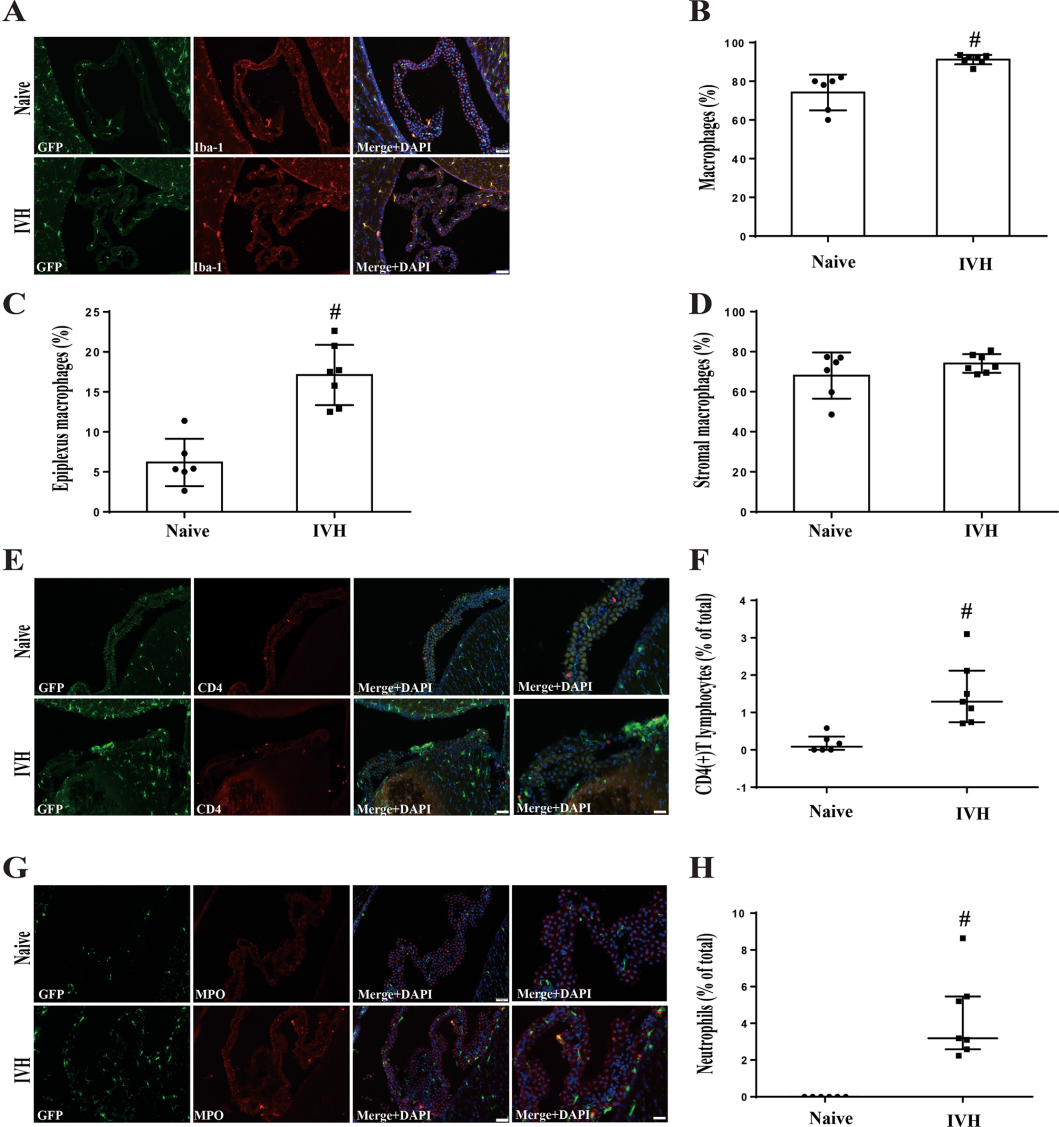
Intraventricular hemorrhage increases the number of ChP macrophages (total and epiplexus), T lymphocytes and neutrophils in CX_3_CR-1^GFP^mice. (**A**) Immunofluorescence staining of ChP Iba-1 in naïve and IVH group at 24 h. Scale bar = 50 μm. (**B**) Quantification of the number of GFP(+)/Iba-1(+) cells as a percentage of the total GFP(+) cells. Values are expressed as mean ± SD, *n* = 6 in naive and *n* = 7 in IVH groups. #*p* < 0.01 by student *t*-test. (**C**) The proportion of epiplexus macrophages to the total number GFP(+) cells was calculated. Values are mean ± SD, *n* = 6 in naive and *n* = 7 in IVH groups. #*p* < 0.01 by student *t*-test. (**D**) The percentage of stromal macrophages relative to the total number of GFP(+) cells was calculated. Values are mean ± SD, *n* = 6 in naive and *n* = 7 in IVH groups. No significant difference between groups by student *t*-test. (**E**) Immunofluorescence staining of ChP CD4 in naïve and IVH groups at 24 h. The first columns scale bar = 50 μm and fourth column scale bar = 20 μm. (**F**) Calculation of the number of T lymphocytes relative to the number of total ChP cells. Values are medians (25th percentile, 75th percentile), *n* = 6 in naive and *n* = 7 in IVH groups. #*p* < 0.01 by Mann-Whitney *U* test. (**G**) Immunofluorescence staining of ChP MPO in naïve and IVH groups at 24 h. The first columns scale bar = 50 μm and fourth column scale bar = 20 μm. (**H**) Estimation of the number of neutrophils relative to the number of total ChP cells cells. Values are median (25th percentile, 75th percentile), *n* = 6 in naive and *n* = 7 in IVH groups. #*p* < 0.01 by Mann-Whitney *U* test

The effects of IVH on different types of immune cells at the ChP was examined. For examples of immune cell identification see Supplemental Fig. 1. Macrophages (GFP(+)/Iba-1(+)) represented a higher proportion of all ChP CX_3_CR1 cells after IVH compared to naïve mice (91.2 ± 2.4 vs. 74.2 ± 9.2% of GFP(+) cells, *n* = 7 & 6; *P* < 0.01, Fig. 3B). Segregating the ChP macrophages into two distinct groups (epiplexus and stromal macrophages) based on their anatomical location showed a marked increase in epiplexus macrophages post-IVH as compared to naïve mice (17.1 ± 3.7 vs. 6.1 ± 2.9%, *n* = 7 &6; *P* < 0.01, Fig. 3C). In contrast, there were no significant difference in stromal macrophages between the two groups.

Furthermore, there was a substantial increase in ChP CD4(+) T lymphocytes following IVH (CD4(+) T lymphocytes (% of total) = 1.29 (0.74, 2.12); *n* = 7) compared to naïve mice (0.08 (0, 0.35); *n* = 6, *P* < 0.01, Fig. 3F). Additionally, while no neutrophils were detected within the ChP of naïve CX_3_CR-1^GFP^ mice (Fig. 3G), there was a substantial increase after IVH (neutrophils (% of total) = 3.19 (2.59, 5.46); *n* = 7, *P* < 0.01 vs. naive, Fig. 3H).

### Intraventricular injection of iron or Prx-2 induced hydrocephalus along with an increase in the total number of GFP(+) cells and an enlargement of their soma size

**Fig. 4:**
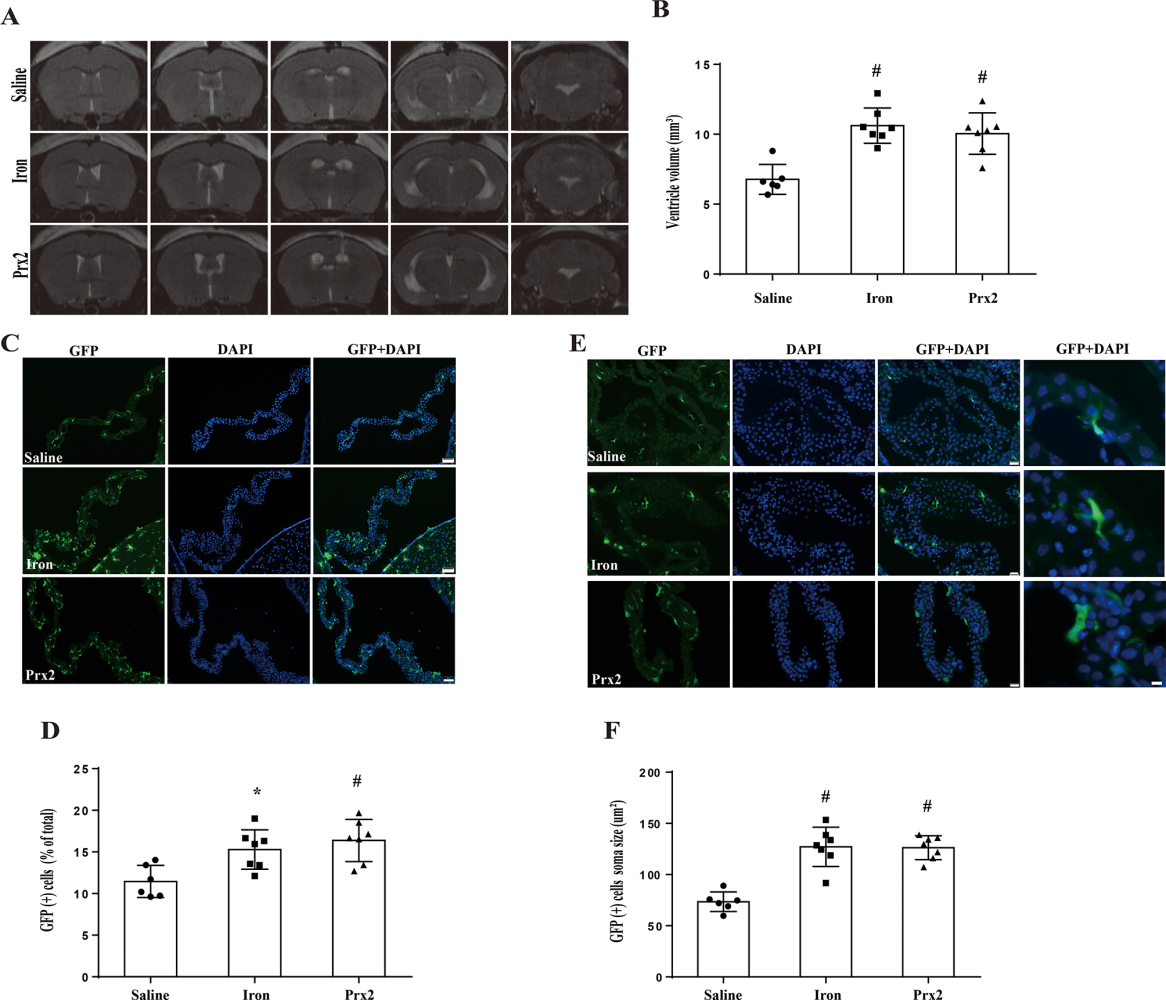
Intraventricular injections of iron and Prx-2 increase ventricular volume, the number and soma size of GFP(+) cells in CX3CR-1^GFP^mice. (**A**) Representative T2-weighted MRI scans 24 h after intraventricular injection of saline, iron and Prx-2 into CX_3_CR-1^GFP^ mice. Note the dilated ventricles in the iron-injected and Prx-2-injected mice. (**B**) Quantification of ventricular volume. Values are means ± SD, *n* = 6 in saline, *n* = 7 in iron and *n* = 7 in Prx-2 groups, #*p* < 0.01 vs. saline group by one-way ANOVA with Tukey’s multiple comparisons test. (**C**) Fluorescence microscopy of GFP(+) cells and DAPI staining in the ChP of mice injected with saline, iron or Prx-2 at 24 h. Scale bar = 50 μm. (**D**) The number of GFP(+) cells was calculated as a percentage of the total number of ChP cells (DAPI). Values are means ± SD, *n* = 6 in saline, *n* = 7 in iron and *n* = 7 in Prx-2 groups. *p < 0.05 for iron vs. saline group and #*p* < 0.01 for Prx-2 vs. saline group by one-way ANOVA with Tukey’s multiple comparisons test. (**E**) Fluorescence microscopy of GFP(+) cells and DAPI staining in the ChP of saline-, iron- and Prx-2-injected mice at 24 h. The first columns scale bar = 20 μm and fourth column scale bar = 5 μm. (**F**) Soma sizes for ChP GFP(+) cells were calculated. Values are means ± SD, *n* = 6 in saline, *n* = 7 in iron and *n* = 7 in Prx-2 groups. #*p* < 0.01 vs. saline group by one-way ANOVA with Tukey’s multiple comparisons test

The effects of two blood components, iron and Prx-2, on hydrocephalus formation and inflammation were examined. Examples of MRIs after intraventricular injection of saline, iron and Prx-2 are shown in Fig. 4A. Ventriculomegaly was induced by intraventricular injection of iron and Prx-2 (ventricle volumes 10.3 ± 0.5 and 10.0 ± 1.4 mm^3^, respectively; *n* = 7) compared to saline injection (ventricle volume 6.7 ± 0.4 mm^3^; *n* = 6, *P* < 0.01, Fig. 4B).

Intraventricular iron injection also increased the number of ChP GFP(+) cells at 24 h (GFP(+) cells were 14.5 ± 1.1% of all ChP cells; *n* = 7) compared to saline injection (11.4 ± 0.7%; *n* = 6, *P* < 0.05, Fig. 4C and D). This was also observed after Prx-2 injection (16.3 ± 2.5%; *n* = 7, *P* < 0.01, Fig. 4C and D). Additionally, the soma size of ChP GFP(+) cells was enlarged following injections of iron (127 ± 7 µm^2^; *n* = 7) and Prx-2 (126 ± 12 µm^2^; *n* = 7) compared to saline (73 ± 4 µm^2^; *n* = 7, *P* < 0.01, Fig. 4E and F).

### Numbers of ChP macrophages (total and epiplexus), T lymphocytes and Neutrophils increased following iron and Prx-2 injection

**Fig. 5:**
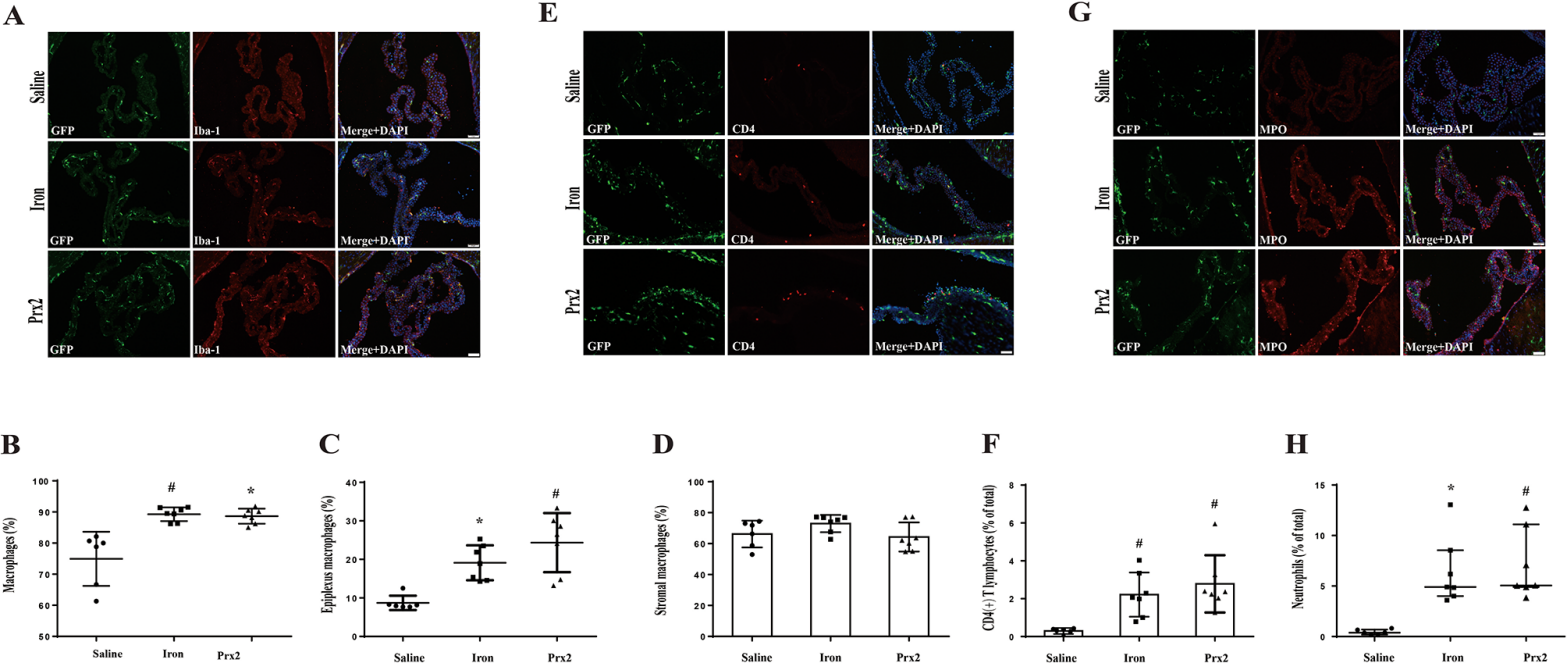
Intraventricular iron or Prx-2 injection increases the number of ChP macrophages (total and epiplexus), T lymphocytes and neutrophils in CX_3_CR-1^GFP^mice. (**A**) Immunofluorescence staining for ChP Iba-1 in saline-injected, iron-injected and Prx-2-injected mice at 24 h. Scale bar = 50 μm. (**B**) The number of GFP(+)/Iba-1(+) cells was calculated as a percentage of the total GFP(+) cells. Values are median (25th percentile, 75th percentile), *n* = 6 in saline, *n* = 7 in iron and *n* = 7 in Prx-2 groups, #*p* < 0.01 for iron vs. saline group and *p < 0.05 for Prx2 vs. saline group by nonparametric Kruskal-Wallis test with Dunn’s multiple comparisons test. (**C**) The percentage of epiplexus macrophages was calculated relative to the total number of GFP(+) cells. Values are median (25th percentile, 75th percentile), *n* = 6 in saline, *n* = 7 in iron and *n* = 7 in Prx-2 groups, *p < 0.05 for iron vs. saline group and #*p* < 0.01 for Prx2 vs. saline group by nonparametric Kruskal-Wallis test with Dunn’s multiple comparisons test. (**D**) The percentage of stromal macrophages was calculated relative to the total number of GFP(+) cells. Values are mean ± SD, *n* = 6 in saline, *n* = 7 in iron and *n* = 7 in Prx-2 groups. No significant differences between groups (one-way ANOVA with Tukey’s multiple comparisons test). (**E**) Immunofluorescence staining of ChP CD4 in mice injected with saline, iron or Prx-2 at 24 h. Scale bar = 50 μm. (**F**) The number of T lymphocytes was calculated relative to the number of total ChP cells. Values are means ± SD, *n* = 6 in saline, *n* = 7 in iron and *n* = 7 in Prx-2 groups. #*p* < 0.01 vs. saline group by one-way ANOVA with Tukey’s multiple comparisons test. (**G**) Immunofluorescence staining of ChP MPO in saline-, iron- and Prx-2-injected mice at 24 h. Scale bar = 50 μm. (**H**) The number of neutrophils was calculated relative to the number of GFP(+) cells. Values are median (25th percentile, 75th percentile), *n* = 6 in saline, *n* = 7 in iron and *n* = 7 in Prx-2 groups. *p < 0.05 for iron vs. saline group and #*p* < 0.01 for Prx2 vs. saline group by nonparametric Kruskal-Wallis test with Dunn’s multiple comparisons test

Our study subsequently explored the response of various ChP immune cells population following intraventricular iron and Prx2 injection. We observed a significant rise in the total number of ChP macrophages (Iba-1(+)) after intraventricular iron injection (89.5% (86.3%, 91.5%) of all GFP(+) cells; *n* = 7, *P* < 0.01) and intraventricular Prx-2 injection (88.8% (86.2%, 90.5%); *n* = 7, *P* < 0.05) compared to naïve mice (79.4% (65.3%, 81.1%); *n* = 6, Fig. 5A and B). Furthermore, when the ChP macrophages were categorized into two groups (epiplexus macrophages and stromal macrophages) according to their anatomical location. It is noteworthy that the percentage of epiplexus cells also increased after intraventricular iron injection (18.9% (14.5%, 23.5%); *n* = 7, *P* < 0.05) and intraventricular Prx-2 injection (26.4% (14.5%, 30.0%); *n* = 7, *P* < 0.01) when compared to intraventricular injection of saline (8.0% (7.6%, 9.3%); *n* = 6, Fig. 5A and C). However, stromal macrophages between these three groups had no significant difference.

In addition, CD4(+) T lymphocytes were markedly increased following iron (% of total = 2.21 ± 1.16; *n* = 7) and Prx-2 injection (2.78 ± 1.51; *n* = 7) compared to saline (0.29 ± 0.15; *n* = 6, *P* < 0.01, Fig. 5E and F). Furthermore, ChP neutrophils surged dramatically after iron (% of total = 4.91 (4.01, 8.54); *n* = 7, *P* < 0.05) and Prx-2 injections (5.05 (4.90, 11.11); *n* = 7, *P* < 0.01) compared to saline controls (0.38 (0.19, 0.69); *n* = 6, Fig. 5G and H).

### Clodronate liposome treatment resulted in a reduction of hydrocephalus, total GFP(+) cell number, soma size, macrophage number

**Fig. 6:**
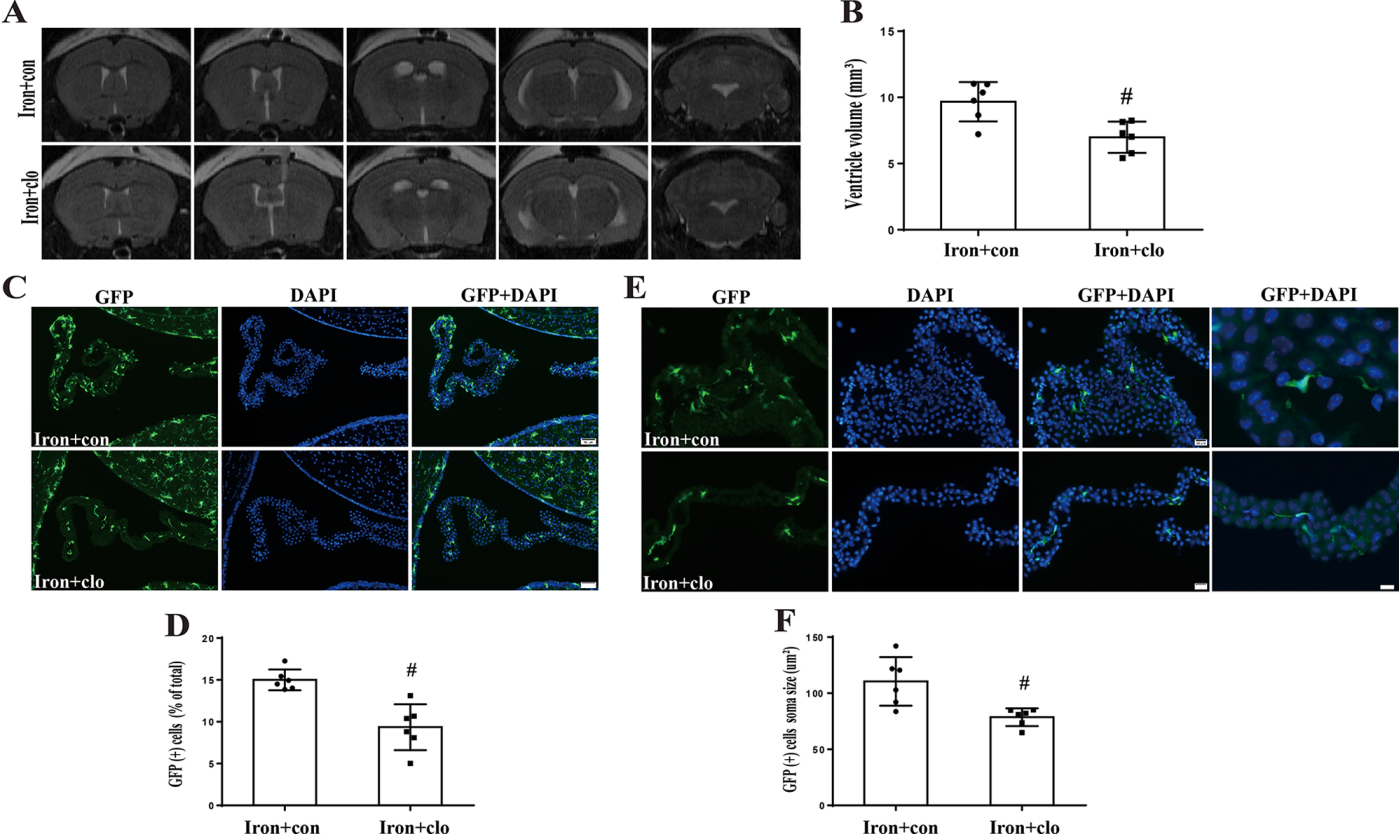
Clodronate treatment decreases ventricular volume, the number of GFP(+) cells and the soma size of GFP(+) cells in CX_3_CR-1^GFP^mice. (**A**) Examples of T2-weighted MRI scans obtained 24 h after intraventricular injection of iron + clodronate liposomes (iron + clo) or iron + control liposomes (iron + con). Note the attenuated ventriculomegaly in the iron + clo group. (**B**) Quantification of ventricular volume. Values are mean ± SD, *n* = 6 in iron + con and iron + clo groups, #*p* < 0.01 by student *t*-test. (**C**) Fluorescence images of GFP(+) cells in the ChP of saline + con-injected and iron + clo-injected mice at 24 h. Scale bar = 50 μm. (**D**) The number of GFP(+) cells was calculated as a percentage of the total number of ChP cells (DAPI). Values are mean ± SD, *n* = 6, #*p* < 0.01 by student *t*-test. (**E**) Fluorescence imaging of GFP(+) cells within the ChP of mice injected with iron + con and iron + clo at the 24-hour time point. The first columns scale bar = 20 μm and fourth column scale bar = 5 μm. (**F**) Soma sizes of ChP GFP(+) cells were quantified. Values are mean ± SD, *n* = 6, #*p* < 0.01 by student *t*-test

We next assessed the clodronate treatment effect on hydrocephalus and relevant underlying mechanisms. Figure 6A showed the MRIs from mice in iron + control group (iron + con) and iron + clodronate group (iron + clo). Notably, clodronate treatment caused a reduction in hydrocephalus (ventricle volume 6.9 ± 0.4 mm^3^ in iron + clo group; *n* = 6) compared to control group mice (ventricle volume 9.6 ± 0.6 mm^3^ in iron + con group; *n* = 6, *P* < 0.01, Fig. 6B). In the iron + clodronate group mice, there was a decrease in the number of total GFP(+) cells at 24 h (9.3 ± 1.1% of all ChP cells; *n* = 6) compared with iron + control group mice (15.0 ± 0. 9.3 ± 1.1%; *n* = 6, *P* < 0.01, Fig. 6C and D). Meanwhile, ChP GFP(+) cell soma size decreased after clodronate treatment in iron + control group (78.6 ± 3.2um^2^; *n* = 6) compared to iron + clodronate group mice (110.5 ± 8.8um^2^; *n* = 6, *P* < 0.01, Fig. 6E and F).

**Fig. 7:**
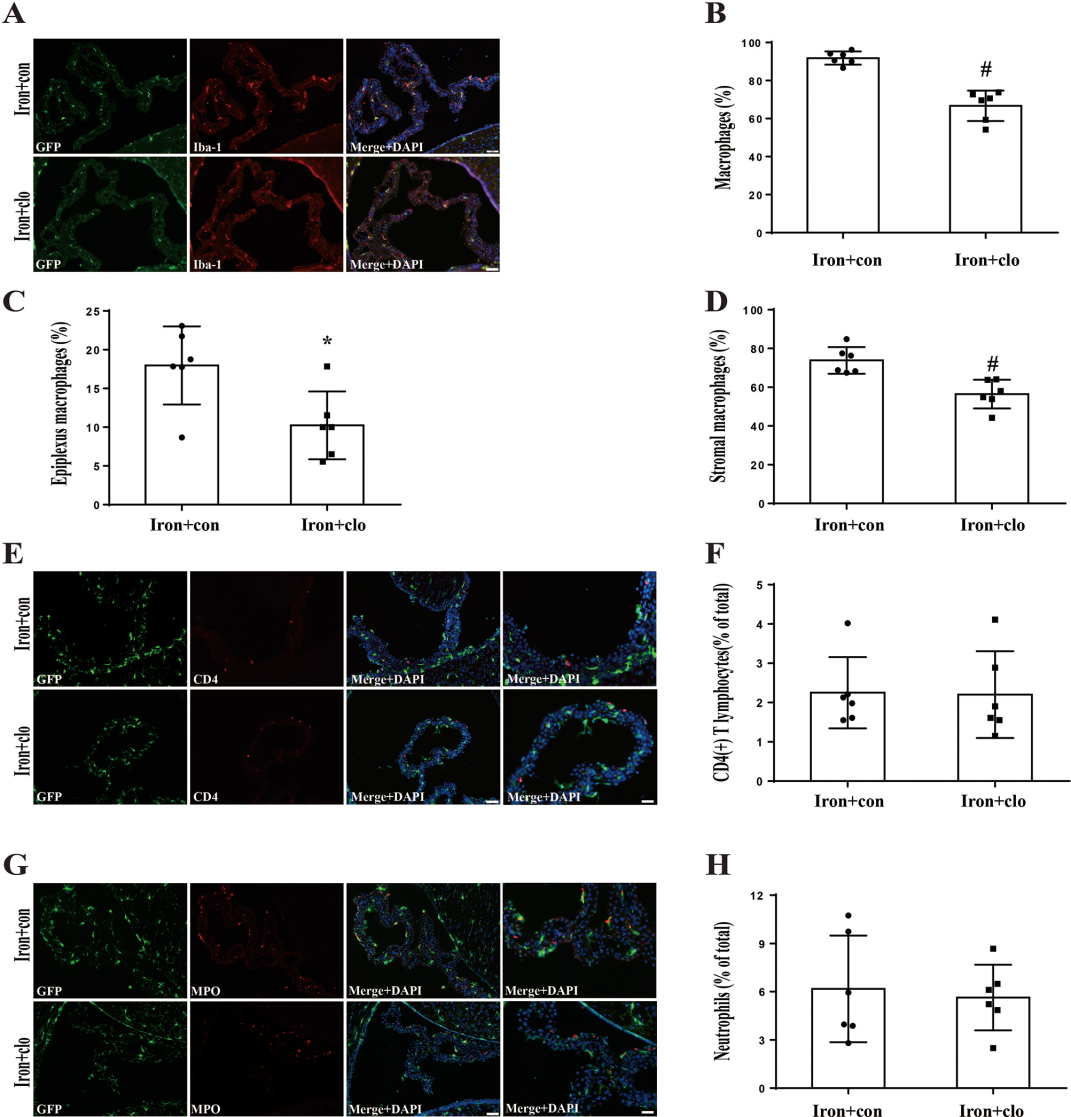
Clodronate treatment reduces ChP macrophages (total, epiplexus and stromal), but not the number of T lymphocytes and neutrophils in CX_3_CR-1^GFP^mice. (**A**) Immunofluorescence staining of ChP Iba-1 in iron + control liposomes (con) and iron + clodronate liposomes (clo) groups at 24 h. Scale bar = 50 μm. (**B**) Calculation of the number of GFP(+)/Iba-1(+) cells (macrophages) as a percentage of total GFP(+) cells. Values are mean ± SD, *n* = 6, #*p* < 0.01 by student *t*-test. The percentage of epiplexus (**C**) and stromal (**D**) macrophages was calculated relative to the total number of GFP(+) cells. Values are mean ± SD, *n* = 6, *p < 0.05 and # *p* < 0.01 by student *t*-test. (**E**) Immunofluorescence staining of ChP CD4 in iron + con and iron + clo group at 24 h. The first columns scale bar = 50 μm and fourth column scale bar = 20 μm. (**F**) The percentage of T lymphocytes was calculated relative to the total number of ChP cells. Values are mean ± SD, *n* = 6, no significant difference by student *t*-test. (**G**) Immunofluorescence staining of ChP MPO in iron + con and iron + clo group at 24 h. The first columns scale bar = 50 μm and fourth column scale bar = 20 μm. (**H**) The percentage of neutrophils was calculated relative to the total number of GFP(+) cells. Values are mean ± SD, *n* = 6, no significant difference by student *t*-test

Subsequently, we assessed the change in ChP macrophages after clodronate treatment in the intraventricular injection iron model. We found that the total number of ChP macrophages decreased after clodronate treatment in iron + control group (91.8 ± 3.4% of all GFP(+) cells; *n* = 6) compared to iron + clodronate group mice (66.7 ± 7.9%; *n* = 6, *P* < 0.01, Fig. 7A and B). When we subdivided the total ChP macrophages into two groups (epiplexus macrophages and stromal macrophages) based on their location. It was evident that the percentage of epiplexs cell also declined after clodronate treatment in iron + control group (17.9 ± 5.0%; *n* = 6) compared to iron + clodronate group (10.2 ± 4.3%; *n* = 6, *P* < 0.05, Fig. 7C). Similarly, stromal macrophages dropped following clodronate treatment in iron + control group (73.8 ± 6.8%; *n* = 6) compared to iron + clodronate group (56.4 ± 7.4%; *n* = 6, *P* < 0.01, Fig. 7D). However, there were no significant differences observed in ChP CD4(+) T lymphocytes and neutrophils between two groups (Fig. 7E-H).

## Discussion

The key findings of this study were: (a) in naïve CX_3_CR-1^GFP^ mice, approximately 75% of GFP(+) cells in ChP are macrophages, with the majority located in the stroma; (b) after IVH and intraventricular injection of iron or Prx-2, both of which induce ventriculomegaly, ChP GFP(+) immune cells increase in number and size; (c) ChP epiplexus (but not stromal) macrophages show significant increases after IVH, iron injection and Prx-2 injection; (d) IVH, iron injection and Prx-2 injection all markedly increase the number of ChP T lymphocytes and neutrophils; (e) when iron was co-injected with clodronate liposomes, ventriculomegaly, ChP immune cell activation and the number of ChP macrophages were all reduced. These results are discussed further below.

Before discussing IVH-induced changes in the ChP immune cell landscape, it is important to consider IVH-related modeling in rodents. The current study used a 30 µl autologous blood injection in mice to mimic an IVH. It also used 12.5 µl injections of saline with and without FeCl_3_ (0.5 mmol/L) and Prx-2 (1 mg/ml), two important erythrocyte components released during cell lysis. 12.5 µl injections were used to reflect the approximate volume of erythrocytes in a 30 µl autologous blood injection. It is important to note that volume of saline injected does not appear to be an important component in the ventricular dilation with FeCl_3_ or Prx-2. In the current study, naïve mice had ventricular volumes of 6.2 ± 0.3 mm³, whereas those given a 12.5 µl intraventricular injection of saline had a ventricular volume of 6.7 ± 0.4 mm³ at 1 day. In a separate unpublished study, we have found a ventricular volume of 6.8 ± 0.2 mm³ at 1 day after a 30 µl intraventricular injection of saline. Thus, by one day, any saline injected has migrated from the ventricles and doesn’t result in ventricular dilation. In contrast, there is evidence that ‘toxic’ effects of erythrocyte components released on lysis contribute to ventricular dilation in IVH. Thus, intraventricular injection of lysed but not intact red blood cells caused ventricular dilation at 1 day in rats, an effect inhibited by an iron chelator deferoxamine [4] and a Prx-2 inhibitor, Conoidin-A [7]. The ventricular dilation induced by IVH (autologous whole blood) was also reduced by deferoxamine in rats [30].

While increasing the volumes of saline up to 30 µl does not cause ventricular dilation in mice, in IVH modeling the amount of blood injected appears important. Thus, in the current study, 30 µl of blood caused an approximate doubling in ventricle size, whereas a recent study by Cao et al. [31] injected 50 µl and trebled ventricular volume. However, it is difficult to dissociate direct volume effects (e.g., injecting different volumes of blood when the aqueduct outflow may be blocked) from there being a greater amount of potential toxic blood components (such as Fe and Prx-2).

Overall, understanding the changes in the ChP immune landscape (including macrophages, neutrophils and T-lymphocytes) after IVH is important for multiple reasons. Macrophages, via phagocytosis, play an important role in hematoma clearance after a cerebral hemorrhage. While this process has been studied extensively in ICH, where monocyte-derived macrophages and resident microglia play important roles [32], much less is known about hematoma phagocytosis in IVH and the role of ChP macrophages. The ChP may either be a source of macrophages or a site of macrophage migration between blood and CSF. Neutrophils are generally thought to enhance CNS injury, including in ICH, via neurotoxic proteins released during degranulation [33]. However, one protein, lactoferrin, released may be beneficial in cerebral hemorrhage because of its iron-binding properties [34]. The role of T-lymphocytes in IVH is unknown but there is evidence that they play an important modulatory role in brain injury after cerebral ischemia, either promoting protection or injury [35]. Leukocytes are a crucial element in the response to pathogens and it is possible that the ChP immune response after IVH may guard against possible pathogen entry into CSF/brain that could occur along with an IVH. Interestingly, Nakano et al. [36] found that one pathogen, Streptococcus mutans, can actually cause cerebral hemorrhage via its expression of a collagen binding protein. In addition, immune cells regulate the function of many cell types via the release of cytokines and chemokines, including recent evidence that they regulate ChP function including CSF secretion [12].

CX_3_CR-1^GFP^ mice, expressing EGFP in macrophages, dendritic cells, NK cells and microglia, are a valuable tool for studying inflammation in various neurological diseases [22]. In this study, using those mice combined with conventional immunohistochemistry, we found macrophages (epiplexus and stromal), dendritic cells, NK cells and lymphocytes, but not neutrophils, in naïve ChP. We observed activation of GFP(+) cells in CX_3_CR-1^GFP^ mouse following IVH without immunofluorescence staining. IVH resulted in an increase in the number (∼ 40%) and size of ChP GFP(+) cells indicating ChP immune cell activation. This activation may be influenced by blood components, such as iron and Prx-2, generated after erythrocytes lysis. Iron has been shown to activate ChP macrophages [3] and Prx-2 can activation ChP macrophages [7] and dendritic cells [8]. Intraventricular injection of iron/Prx-2 mimicked the effects of IVH, increasing the number of ChP GFP(+) immune cells (∼ 30–40%) and their soma size suggesting they may play important roles in ChP immune cell activation after IVH. The ChP functions as an immunological hub, housing various types of peripheral immune cells within its stroma, such as dendritic cells, macrophages, and T cells. Together with the epithelial cells, these immune cells engage in immunosurveillance, identifying pathogens and alterations in the cytokine environment. Upon activation, they secrete homing molecules that stimulate the chemotaxis of circulating immune cells, thereby orchestrating an immune response at the ChP [10].

In using CX_3_CR-1^GFP^ mice to study the PHH it is important to make sure that these mice have a similar response to IVH and blood components. The degree of ventriculomegaly after IVH and ventricular iron and Prx-2 injection in CX_3_CR-1^GFP^ mice was similar to that previously reported in rodents [3, 8, 28].

To further investigate the changes to the ChP immune cell landscape after IVH and blood component injection, the ChP of CX_3_CR-1^GFP^ mice was further probed with traditional immunohistochemistry. Iba-1 antibody was used to distinguish macrophages from other ChP GFP(+) immune cells. GFP(+)/Iba-1(+) cells were defined as macrophages and subdivided into epiplexus and stromal macrophages based on their location relative to the ChP epithelium [28, 37, 38]. Macrophages were the predominant (∼ 75% of GFP(+) cells) cell type at the ChP expressing GFP in naïve mice with the majority being stromal. There were fewer dendritic and NK cells. After IVH, an even greater % percentage of GFP(+) cells were macrophages (∼ 90%) and this was also the case after intraventricular iron or Prx-2 injection.

Macrophages play a crucial role in inflammatory responses and can produce various proinflammatory cytokines, including interleukin-1β (IL-1β), -6, -12, -23, -10 and tumor necrosis factor-a [39, 40]. Proinflammatory cytokines, especially IL-1β, are potent drivers of leukocyte recruitment to the CNS. The number of epiplexus macrophages increased significantly following IVH and intraventricular injection of iron/Prx-2. Interesting, IVH and intraventricular injection of iron/Prx-2 did not significantly change stromal macrophage numbers. Our prior study, however, illustrated that intraventricular iron injection led to hydrocephalus and an increased number of stromal macrophages in both aged and young rats [3]. This discrepancy might be attributed to species or model differences. Being on the apical surface of the ChP epithelium, epiplexus cells are situated to respond the intraventricular blood or factors released from the hemorrhage (such as iron and Prx-2) into CSF.

There are several possible explanations for the increase in epiplexus macrophages following IVH and intraventricular iron/Prx-2 injection. The first is blood-derived monocyte migration. Ge et al., found that blood-derived monocyte infiltrated from blood into ChP and CSF after ischemic stroke [41]. Second, stromal macrophages and dendritic cells can migrate to the apical surface of epithelium [8]. Third, macrophage proliferation could underlie the increase in epiplexus cells. An increase Ki67 (proliferation marker) positive ChP macrophages has been reported in PHH rats [12].

T lymphocytes were identified using CD4 immunohistochemistry in this study. In naïve mice, they were rare compared to total ChP cells (ratio 1:1000). However, IVH resulted in a marked (∼ 16-fold) increase in ChP T lymphocyte numbers, and intraventricular injections of iron and Prx-2 replicated this effect. These findings suggest that iron and Prx-2 may be involved in T lymphocyte infiltration into ChP and possibly CSF following IVH. Lymphocyte trafficking across blood-choroid plexus barrier has been reported in various conditions, including ischemic stroke [42], feline immunodeficiency virus infection [43] and ventricular injection of tumor necrosis factor-alpha [44]. The blood-brain barrier regulates lymphocyte traffic into the CNS, maintaining its barrier properties and selectively permitting the passage of activated T lymphocytes through cerebral vessels in vivo [45]. Activated auto-aggressive CD4 (+) T lymphocytes, which are initially activated outside the CNS, can migrate into the CNS and trigger cellular events that result in edema, inflammation, and demyelination within the CNS white matter [46]. In vivo studies provide evidence that immunocompetent cells, such as T lymphocytes, can infiltrate the healthy CNS regardless of their antigen specificity [47]. The ChP can also promote T cell trafficking in response to inflammation, influencing adaptive immunity in the CNS [48]. To our knowledge, this study is the first to report T lymphocyte infiltration into the ChP after IVH, and further research is needed to elucidate the function of these lymphocytes in IVH and PHH.

Neutrophils within ChP were identified using MPO antibody. ChP neutrophils were absent in naïve mice. However, IVH and intraventricular injection of iron or Prx-2 resulted in a marked increase in ChP neutrophils (neutrophils: total ChP cells ratios of ∼ 3–5:100). These results suggest that iron and Prx-2 may play a role in neutrophil infiltration following IVH. Previous studies have demonstrated that intraventricular injection of Prx-2 [7] and thrombin [16] results in the accumulation of neutrophil within the ChP. Neutrophils serve as a primary defense mechanism within the innate immune system, combatting external microbial threats [49]. The origin of the neutrophils found in the ChP, whether they migrate across the ChP blood vessel or the ventricular wall, remains unclear. Nonetheless, accumulating evidence suggests that neutrophils can also contribute detrimentally to intracerebral injury through the release of extracellular traps [50, 51]. However, the signaling mechanisms and specific pathways through which blood components (iron, Prx-2, thrombin) activate neutrophils and recruit them to the ChP and potentially CSF require future investigation.

Evidence indicates that the activation of inflammatory pathways at the ChP can induce CSF hypersecretion, which may contribute to hydrocephalus formation after IVH [11]. Recent study has revealed that PHH and post-infectious hydrocephalus is characterized by TLR4-mediated ChP immune cell signaling and SPAK-dependent ChP transepithelial ion transport [12]. Reduction of macrophages adhering to the ChP epithelium and stroma attenuates ChP inflammation, suggesting that macrophages reduction may reduce CSF secretion by the ChP epithelium and ultimately decrease ventricle dilation. The present study supports this hypothesis by using clodronate liposomes to deplete ChP macrophages. Liposomes containing clodronate induce apoptosis in macrophages, leading to their self-elimination, and have been utilized for macrophage depletion in various tissues [52]. When macrophages engulf these clodronate-loaded liposomes, the liposomes are broken down by lysosomal phospholipases, releasing clodronate into the cells’ interior, which then triggers apoptosis through inhibits the adenosine triphosphate production enzyme adenine nucleotide translocase [53]. Clodronate liposome treatment reduced ventriculomegaly induced by intraventricular iron injection by reducing the number of ChP macrophages (both epiplexus and stromal macrophages). In contrast to macrophage reduction, clodronate liposomes did not impact the number of ChP T lymphocytes and neutrophils within ChP. Targeting macrophages with clodronate liposomes has also reduced ventriculomegaly induced by intraventricular injection of Prx-2 in rats [8]. Together, this suggests that targeting ChP macrophage activation might be a therapeutic target in PHH.

Several limitations should be noted in this study. It primarily serves as a proof-of-concept study that identifies alterations in ChP immune cells (macrophages, lymphocytes and neutrophils) following IVH and intraventricular injection of blood components (iron and Prx-2), as well as the associated inflammatory responses in PHH and iron/Prx-2-induced acute (24 h) ventriculomegaly. Long-term changes were not examined and functional outcomes were not assessed. The results may not be applicable to both sexes, as only male CX_3_CR-1^GFP^ mice were studied. While the results indicate the clodronate liposomes deplete ChP macrophages but not lymphocytes and neutrophils, it remains uncertain whether the nonspecific uptake of clodronate liposomes by other cells might influence hydrocephalus formation and the inflammatory response.

In summary, IVH and blood components (iron, Prx-2) cause marked changes in ChP immune landscape. Reduction of ChP macrophages through clodronate liposome treatment depletes ChP macrophages and attenuates iron-induced ventriculomegaly. This suggests that the ChP immune cells response may be a potential therapeutic target in IVH-induced hydrocephalus.

### Electronic supplementary material

Below is the link to the electronic supplementary material.

Supplementary Material 1

## Data Availability

The datasets used and/or analyzed in this study are available from the corresponding author upon reasonable request.

## Abbreviations

IVH: Intraventricular hemorrhage
SAH: subarachnoid hemorrhage
ICH: intracerebral hemorrhage
PHH: post-hemorrhagic hydrocephalus
CSF: cerebrospinal fluid
Prx-2: Peroxiredoxin-2
DAMP: damage-associated molecular pattern
ChP: choroid plexus
CNS: central nervous system
BAMs: border-associated macrophages
NK cells: natural killer cells
EGFP: enhanced green fluorescent protein
MRI: magnetic resonance imaging
MPO: myeloperoxidase
SD: standard deviation
