# Supplementary figures and images for: Choroid plexus immune cell response in murine hydrocephalus induced by intraventricular hemorrhage
Source: Fluids Barriers CNS. 2024 Apr 23;21:37. doi: 10.1186/s12987-024-00538-4 (PMC11036653; doi:10.1186/s12987-024-00538-4)

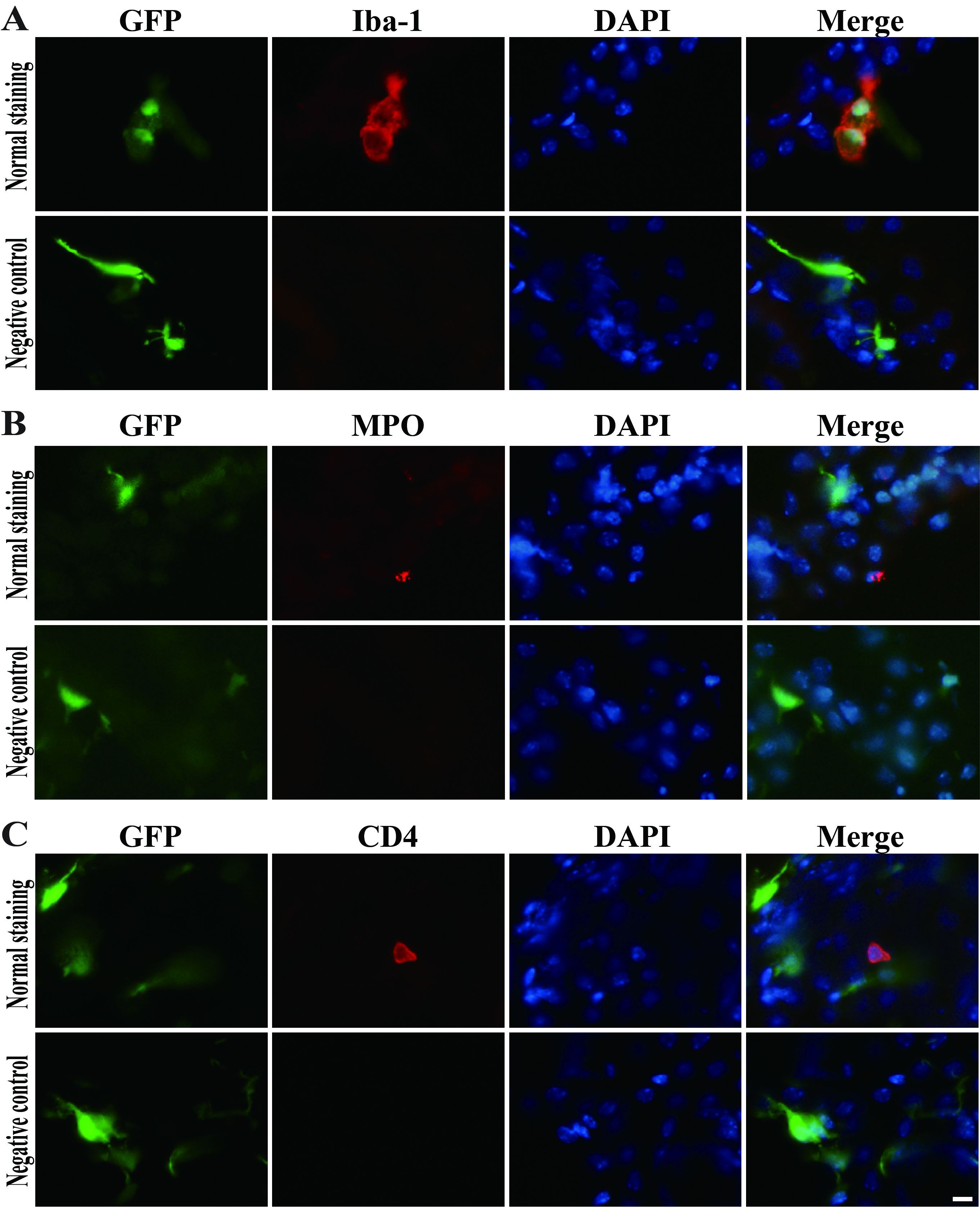

Supplement: Supplementary file 1 — Supplementary Material 1 [file 12987_2024_538_MOESM1_ESM.jpg]
